# Combination of post-fascia iliaca compartment block and dexmedetomidine in pain and inflammation control after total hip arthroplasty for elder patients: a randomized control study

**DOI:** 10.1186/s13018-020-1562-6

**Published:** 2020-02-10

**Authors:** Xiaofen Liu, Xianwen Hu, Rui Li, Ye Zhang

**Affiliations:** Department of Anesthesiology and Perioperative Medicine, The Second Hospital of Anhui Medical University, No. 678, Furong Road, Hefei, 230000 China

**Keywords:** Fascia iliaca compartment block, Dexmedetomidine, Pain, Inflammation, Total hip arthroplasty

## Abstract

**Objective:**

This study aims to investigate the efficiency of fascia iliaca compartment block (FICB) combined with dexmedetomidine (DEX) in postoperative and inflammation management for elder patients after total hip arthroplasty.

**Methods:**

The present randomized controlled study included a total of 119 elder patients who received total hip arthroplasty in our hospital from March 2016 to December 2018. These patients were divided into three groups: control group, patients received routine general anesthesia; FICB group, patients received additional FICB after surgery; and combined group, patients received both pre-treatment of DEX and post-treatment of fascia iliaca compartment block. The serum levels of interleukin (IL)-1β, IL-6, and C-reactive protein (CRP) were measured by ELISA. The visual analog scale (VAS) score was measured at 12, 24, 48, and 72 h after surgery, and the patient-controlled intravenous analgesia (PCIA) pressing time within 48 h after surgery was also recorded. The Pittsburgh sleep quality index (PSQI) was used to measure the sleep quality before and at 1 month after surgery.

**Results:**

The VAS scores were significantly lower in patients in the combined group, when compared with the other two groups, at 12, 24, 48, and 72 h after surgery. In addition, the VAS scores at all time points were significantly lower in the FICB group than the control group. The PCIA pressing times were also remarkably lower in the combined group. At 4, 24, 48, and 72 h after surgery, the serum levels of these inflammatory factors were the lowest in the combined group, and the PSQI scores were significantly lower in the combined group, when compared with the other two groups, while the control group had the highest PSQI scores among the three groups. There was no severe side effects and significant difference observed.

**Conclusion:**

FICB combined with DEX reduced the postoperative pain, improved the sleep condition, and decreased the serum levels of inflammatory factors after total hip arthroplasty.

## Introduction

Hip fractures are common in elder patients with osteoporosis, which result in a high rate of morbidity, reduced quality of life, and long-term care services [1–4]. Total hip arthroplasty (THA) is a widely adopted surgical method for end-stage hip fractures [5–7]. It has been estimated that approximately 572,000 cases may need THA in 2030 in the USA and that over 6.26 million patients may suffer from hip fractures in 2050 [8–10]. However, despite the wide application of THA for femoral fractures, the postoperative pain management remains as a clinical challenge.

Analgesic methods used in THA usually include the application of anesthetics combined with nerve block methods [11, 12]. In a meta-analysis, the authors reported that both the femoral nerve block and fascia iliaca block could reduce the pain for patients after total knee and hip arthroplasty [11]. The fascia iliaca compartment block (FICB) is a newly developed method and is mainly used in lower limb surgeries [13]. It has been reported that FICM has superior analgesic effects in surgeries for hip fractures and hip flexion [14, 15]. It was also found that the FICB could reduce the incidence of delirium in intermediate risk patients [16]. However, most studies have used the FICB as a preoperative treatment, and the application of postoperative FICB remains inadequately reported. Dexmedetomidine (DEX), which is a kind of α2-adrenergic receptor agonist, is mainly used for pain management during or after surgery [17]. It has been reported that the DEX has sedative and analgesic properties and that this could also protect patients from postoperative cognitive dysfunction [18, 19].

However, to date, few studies have reported the combined use of DEX and FICB after THA. In the present prospective study, a randomized controlled study was conducted to investigate the efficiency of FICB combined with DEX in postoperative treatment and inflammation management for elder patients after THA. The present study might provide more clinical evidences for the application of the combined use of FICB and DEX in hip surgeries.

## Methods and materials

### Patients

The present randomized controlled open study included a total of 119 elderly patients (≥ 60 years old), who received THA in our hospital from March 2016 to December 2018. All patients who met the inclusion criteria were consecutively enrolled during the study period. The inclusion criteria were as follows: patients who were diagnosed with hip fracture and received THA, patients ≥ 60 years old, and patients with an American Society of Anesthesiologists (ASA) score of I–III. The diagnosis of hip fracture was confirmed by both X-ray and computed tomography (CT) scan. All surgeries adapted the anterior midline approach. The exclusion criteria were as follows: patients with other fractures; patients with other severe system diseases, such as severe liver, renal, or heart diseases; patients who received analgesics, such as cyclooxygenase inhibitors and opioid receptor agonists, within 1 month before the study; patients with insomnia; and patients with chronic inflammation or pain. All patients provided a signed informed consent. The present study was approved by The Second Hospital of Anhui Medical University.

### Anesthesia strategy

All patients were randomly divided into three groups using a computer-generated list by the SPSS software through a third physician: (1) control group (*n* = 39), patients only received routine general anesthesia; (2) the FICB group (*n* = 40), patients received an additional FICB after surgery; and (3) combined group (*n* = 40), patients received both the pre-treatment of DEX and post-treatment of FICB. For anesthesia induction, these patients were intravenously injected with 1.5 mg/kg of propofol, 0.03 mg/kg of midazolam, 0.6 mg/kg of rocuronium, and 1 μg/kg of remifentanil, followed with endotracheal intubation and mechanical ventilation. Then, 4 mg/kg of propofol and 0.2–0.5 μg/(kg min) of remifentanil were used for anesthesia maintenance.

For the treatment of DEX, 0.6 μg/kg/h of DEX was maintained by intravenous injection after anesthesia induction until 30 min before the end of the surgery, and the other two groups received 0.6 μg/kg/h of 0.9% NaCl. For the FICB, the FICB was conducted under ultrasound guidance within 30 min after surgery. Briefly, the iliopsoas, iliac fascia, and fascia lata were observed under ultrasound, and a peripheral nerve plexus stimulation needle was inserted into the iliac fascia to inject 30 ml of 0.2% ropivacaine. All patients received patient-controlled intravenous analgesia (PCIA) containing 0.12% bupivacaine + sufentanil citrate of 0.02 μg/kg/h, which were dissolved in 150 ml of normal saline. The single dose of PCIA was limited to 1 ml, with a continuous infusion rate 4 ml/h, and the drug locking time was set to 15 min. The routine low molecular weight heparin calcium anticoagulant therapy and antibiotic prevention of infection were performed for all patients.

### Measurement of inflammatory factors

Briefly, peripheral blood samples (5 ml) were collected before the surgery and at 24 h after surgery. The serum levels of IL-1β (Cat. no. ab46052, Abcam), IL-6 (Cat. no. ab178013, Abcam), and CRP (Cat. no. LS-F26721, Lifespan Bio.) were determined using commercial enzyme-linked immunosorbent assay (ELISA) kits.

### Data collection

The patient characteristics, including age, gender, body mass index (BMI), and fracture type, were recorded. The resting and moving visual analog scale (VAS) scores were all measured at 12, 24, 48, and 72 h after surgery. The PCIA pressing time within 48 h after surgery was also recorded. The Pittsburgh sleep quality index (PSQI) was used to measure the sleep quality before and at 1 month after surgery [20]. The side effects within 3 days after surgery were also recorded.

### Statistical analysis

The measurement data was expressed as mean ± standard deviation (SD) for continuous data. Counting materials were compared using chi-square test. The comparison among three or more groups were conducted using one-way analysis of variance (ANOVA), followed by Tukey post hoc test. *P* < 0.05 was considered statistically significant. All calculations were performed using SPSS 20.0.

## Results

### Characteristics of all patients

**Fig. 1:**
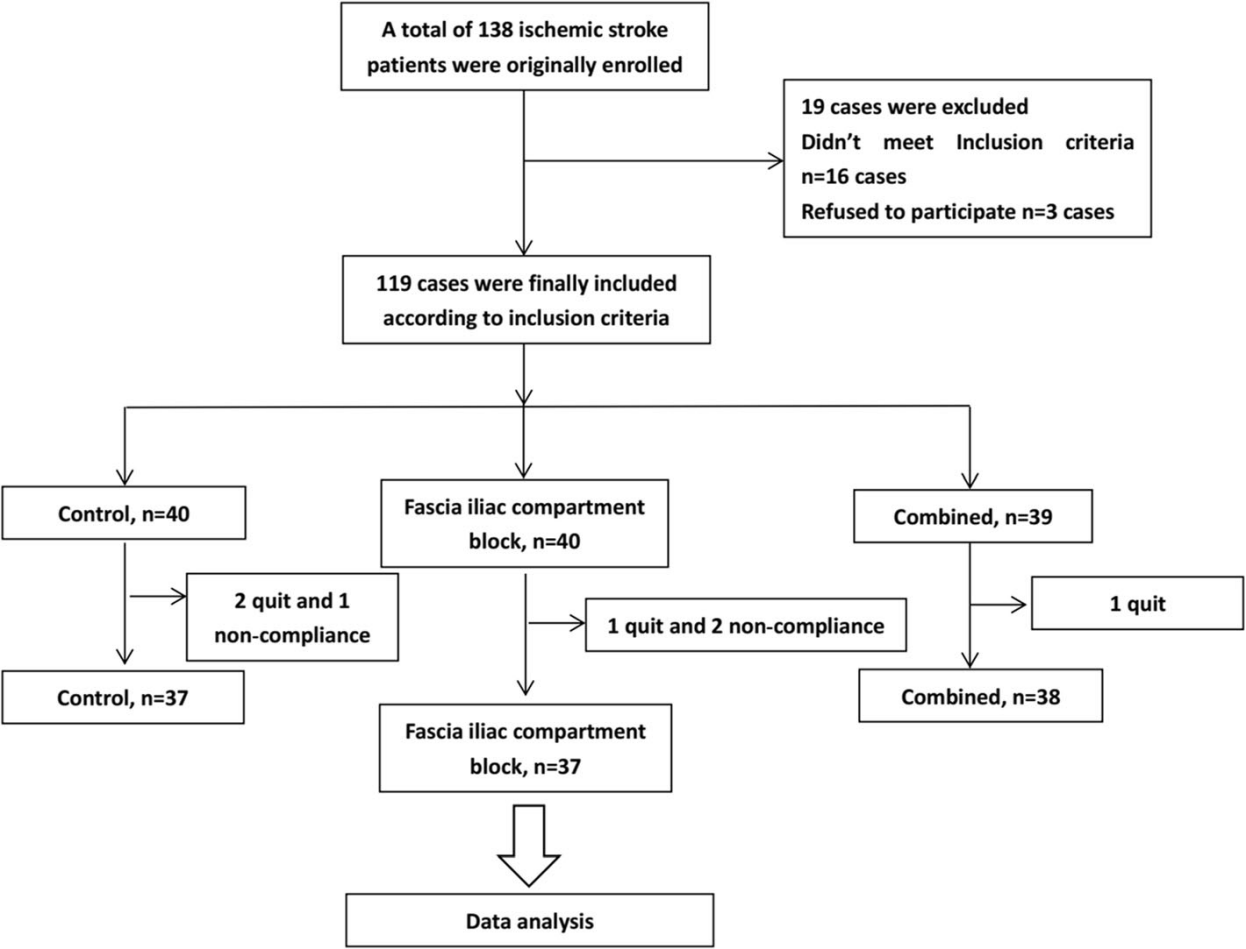
The flowchart

During the study period, four patients quit the study, while three patients were excluded due to bad compliance. Finally, 112 patients were maintained, with 37 patients in the control group, 37 patients in the FICB group, and 38 patients in the combined group (Fig. 1). Among all patients, 47 patients had intertrochanteric fracture of the femur, while 65 patients had fracture of the femoral neck. However, no significant difference was found for age, gender, BMI, and fracture type among the different groups of patients (Table 1).

**Table 1:** Patients characteristics

Variables	Control, *n* = 37	FICB, *n* = 37	Combined, *n* = 38	*P* value
Age, year	70.00 ± 5.69 (62~79)	70.05 ± 5.52 (60~78)	67.37 ± 6.21 (60~79)	0.077
Gender, female (%)	12 (32.43)	11 (29.73)	13 (34.21)	0.792
BMI (kg/m^2^)	23.05 ± 2.89	23.24 ± 3.13	22.81 ± 3.02	0.828
Fracture type, *n* (%)				0.926
Fracture of femoral neck	21 (56.76)	22 (59.46)	22 (57.89)	
Intertrochanteric fracture of femur	16 (43.28)	15 (40.54)	16 (42.11)	

*BMI* body mass index

### The combined use of DEX and FICB in pain condition after THA

**Fig. 2:**
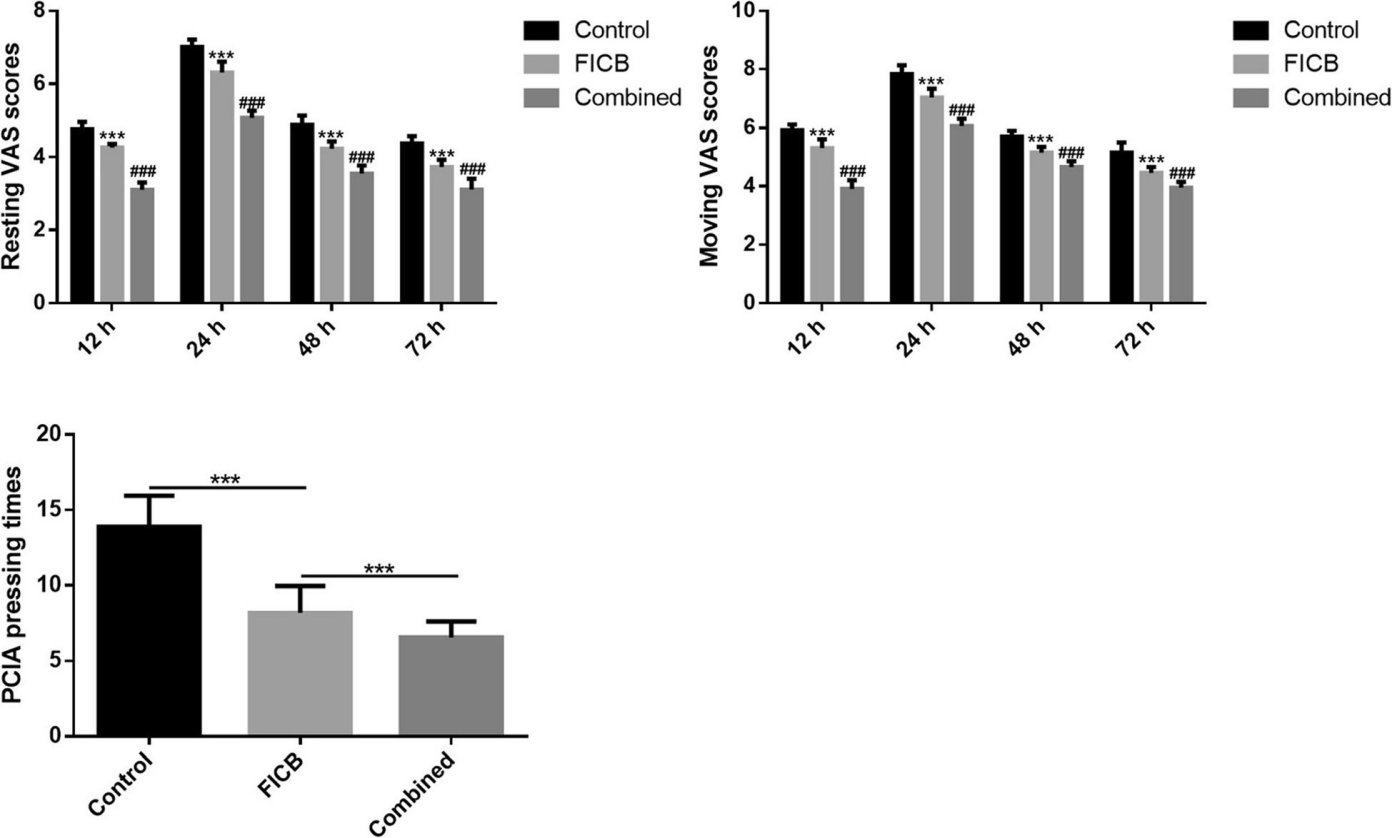
The resting and moving VAS scores and PCIA pressing times in different groups of patients. ***P < 0.05 vs. control, ^###^*P* < 0.05 vs. FICB. VAS, visual analog scale; PCIA, patient-controlled intravenous analgesia

The pain condition for the different groups of patients was determined by the VAS score and PCIA pressing times. As shown in Fig. 2, both the resting and moving VAS scores were significantly lower in patients of the combined group when compared with the other two groups at 12, 24, 48, and 72 h after surgery (*P* = 0.000, for all comparisons within the three groups at all points). Furthermore, the VAS scores at all time points were significantly lower in the FICB group when compared with the control group (*P* = 0.000, for all comparisons within the three groups at all points). Similar results were also observed for PCIA pressing times within 48 h after surgery.

### Effect of the combined use of DEX and FICB on inflammatory factors after THA

The inflammatory factors before and at 24 h after surgery were determined. The results revealed that the serum levels for all factors were significantly upregulated at 24 h after surgery for all groups when compared with the levels before surgery. Meanwhile, at 24 h after surgery, the serum levels of these inflammatory factors were the lowest in the combined group, when compared with the other two groups, and the difference was statistically significant (*P* = 0.000, for all comparisons; Table 2). Patients in the control group exhibited remarkably higher serum levels of inflammatory factors, when compared with the combined group and FICB group (*P* = 0.000, for all comparisons).

**Table 2:** Effect of combination use of DEX and fascia iliaca compartment block on inflammatory factors after total hip arthroplasty

Variables		Control, *n* = 37	FICB, *n* = 37	Combined, *n* = 38
IL-6, pg/ml	Before	84.89 ± 2.88	84.26 ± 3.39	85.64 ± 2.90
	24 h	173.45 ± 12.04	163.48 ± 6.90^a^	137.62 ± 8.18^a,b^
IL-1β, pg/ml	Before	34.34 ± 2.94	34.33 ± 3.18	34.99 ± 2.91
	24 h	54.15 ± 3.05	50.22 ± 3.32^a^	45.50 ± 2.87^a,b^
CRP, mg/L	Before	7.51 ± 0.92	7.63 ± 0.83	7.52 ± 0.74
	24 h	41.53 ± 1.38	36.45 ± 2.26^a^	34.41 ± 2.59^a,b^

*IL* interleukin, *CRP* C-reactive protein

^a^*P* < 0.05 vs. control

^b^*P* < 0.05 vs. FICB

### Effect of the combined use of DEX and FICB on sleep quality index and the side effects

Lastly, the combination of DEX and FICB on sleep quality index and the side effects were measured. As shown in Table 3, the PSQI scores were significantly lower in the combined group than in the other two groups, while patients in the control group had the highest PSQI scores among the three groups (*P* = 0.000). The side effects in all groups were also recorded, and no severe side effects or significant difference was observed.

**Table 3:** Effect of combination use of DEX and fascia iliaca compartment block on sleep quality index and the side effects

Variables	Control, *n* = 37	FICB, *n* = 37	Combined, *n* = 38	*P* value
PSQI before	12.08 ± 1.64	11.86 ± 1.29	12.00 ± 1.34	0.806
PSQI after	10.24 ± 1.23	7.76 ± 1.48	6.00 ± 1.47	0.000
Side effects, *n* (%)				0.731
Nausea	2 (5.4)	1 (2.7)	1 (2.6)	
Emesis	1 (2.7)	1 (2.7)	2 (5.2)	
Pruritus	1 (2.7)	1 (2.7)	1 (2.6)	

*PSQI* Pittsburgh sleep quality index

## Discussion

Pain management after THA is a main problem that affects a patient’s postoperative recovery. At present, several strategies have been reported to enhance the postoperative recovery and reduce the pain, such as oral and intramuscular analgesics, local block at the operative site, nerve block, intravenous controlled analgesia, and epidural controlled analgesia [21, 22]. Among these methods, the FICB is a newly developed method, in which local anesthetics are injected into the FICB and is mainly used in surgeries for the lower limbs [16]. Although several studies have reported the application of FICB for lower limb surgeries, few studies have focused on the combined use of the FICB and DEX in THA. In the present study, it was demonstrated that the combination of FICB and DEX could reduce postoperative pain, improve sleep condition, and decrease serum inflammatory factors after THA.

The FICB has been used in several types of surgeries. Yu et al. compared a continuous femoral nerve block and a continuous fascia iliaca compartment block in THA surgery for elder patients and found that the continuous fascia iliaca compartment block has a better effect on pain condition [23]. Williams et al. revealed that standard analgesia combined with FICB significantly reduced the VAS scores of patients with femoral neck fractures after surgery [24]. In a meta-analysis, Steenberg et al. reported that the FICB was better than opioids during movement and has lower preoperative analgesia consumption, a longer time for first request, and a reduced time to perform the spinal anesthesia [13]. In the present study, it was also found that the FICB treatment reduced postoperative pain, improved the sleep condition, and decreased the serum inflammatory factors. Inflammatory factors, such as IL-1β, IL-6, and CRP, have also been reported to be elevated in surgeries, including THA. In an earlier study, it was demonstrated that the levels of IL-6 and TNF-α increased after THA [25]. It was also reported that CRP levels remained high during the entire observational period after surgery for THA [26]. In addition, studies have found that DEX could also reduce the levels of IL-1β, IL-6, and CRP, both in vivo and in vitro [27–29]. All these results were consistent with the present findings.

DEX is presently widely used for pain postoperative management in various surgeries, including THA. It has been reported that preoperative intravenous DEX can prevent tourniquet-induced hypertension in orthopedic operations [30]. In a case report, it was reported that DEX was effective as a sedative and analgesic for a total hip replacement patient [31]. In a recent research, Mei et al. reported that the intraoperative sedation of DEX was better than propofol in THA for elder patients [32]. In addition, the combined use of DEX and other block anesthetic methods have also been reported in several studies. It was reported that DEX could be used as an adjuvant to 2% lignocaine in an infraorbital nerve block [33]. Another study also revealed that DEX could reduce the morphine consumption, VAS scores, and incidence of postoperative nausea/vomiting [34]. In the present study, it was found that DEX could enhance the pain measurement effects of the FICB. Furthermore, this also reduced the serum levels of inflammatory factors and improved the sleep condition, with no obvious side effects. However, the present study has some limitations. First, the study sample size was limited. Second, all patients were from a single center. Third, the long-term effects of the method were not investigated. Hence, further studies are needed to confirm these results.

## Conclusion

In conclusion, a prospective randomized control study was conducted to investigate the efficiency of FICB combined with DEX after THA. It was observed that the FICB combined with DEX reduced the postoperative pain, improved the sleep condition, and decreased the serum levels of inflammatory factors after THA. The present study might provide more clinical evidences for the application of the FICB and DEX in the postoperative treatment of THA.

## Abbreviations

CRP: C-reactive protein
DEX: Dexmedetomidine
ELISA: Enzyme-linked immunosorbent assay
FICB: Fascia iliaca compartment block
PCIA: Patient-controlled intravenous analgesia
PSQI: Pittsburgh sleep quality index
THA: Total hip arthroplasty
VAS: Visual analog scale
